# Physicochemical and Emulsifying Properties of *Melia azedarach* Gum

**DOI:** 10.1155/2024/3308441

**Published:** 2024-08-22

**Authors:** Kofi Acheampong Asamoa Mensa, Noble Kuntworbe, Yaa Asantewaa Osei, Mariam El Boakye-Gyasi, Frederick William Akuffo Owusu, Lawrence Michael Obeng, Osei-Asibey Antwi, Winifred Naa Adoley, Kwabena Ofori-Kwakye

**Affiliations:** Department of Pharmaceutics Faculty of Pharmacy and Pharmaceutical Sciences Kwame Nkrumah University of Science and Technology, Kumasi, Ghana

## Abstract

Naturally occurring hydrophilic colloids are versatile excipients in drug delivery systems. They are often used as coating materials, disintegrating agents, binders, emulsion stabilizers, and other applications. This study sought to investigate the physicochemical and emulsifying properties of gum extracted from *Melia azedarach* (MA). The gum was harvested, authenticated, and purified using ethanol precipitation. Physicochemical, microbial, and proximate analyses were performed on the purified gum. Oil of olive emulsions containing different amounts (5–15%w/v) of the gum as emulsifiers were prepared by homogenization. The zeta potential, creaming index, and average droplet size of products were assessed. The effects of pH changes, temperature, and monovalent and divalent electrolytes on the stability of the emulsions were also investigated. The yield of the gum after purification was 68.3%w/w. The gum has low moisture content and good swelling properties. Lead, copper, cadmium, and mercury were not detected. Emulsions containing 15%w/v of acacia or MA gum had the smallest average (Z-average) droplet size (acacia: 1.837 ± 0.420 *μ*m; MA gum: 2.791 ± 0.694 *μ*m) and the highest zeta potential (acacia: −30.45 mV; MA gum: −32.867 mV). Increasing the concentration of the gums increased the emulsion viscosity with MA gum emulsions being more viscous than corresponding acacia emulsions. MA gum emulsions had higher emulsion capacity and stability but lower creaming index relative to acacia gum emulsions of similar concentrations. Potassium chloride (KCl) reduced zeta potential but increased Z-average for emulsions prepared with either gum. Calcium chloride (CaCl_2_) produced a similar but more pronounced effect. When the pH was decreased from 10 to 2, the zeta potential of the droplets was reduced, but the droplet size of emulsions prepared from either gum was increased. Increasing temperature from 25 to 90°C produced no significant (*p* value >0.9999) change in droplet size. These findings suggest that MA gum is a capable emulsifying agent at 15%w/v.

## 1. Introduction

Synthetic emulsifying agents are commonly used to stabilize pharmaceutical emulsions. Though effective stabilizers, they are costly, produce toxic effects through direct or indirect means, and cause environmental problems [1]. Most of these synthetic surfactants affect the disposition of encapsulated drugs. Anionic surfactants may interact with proteins and enzymes, altering them and rendering them defective [2].

Natural proteins and polysaccharides used in food emulsions such as milk, dressings, and ice cream are nontoxic, nonirritant, and biocompatible [3]. Gums disperse in water to yield highly hydrated particles of colloidal dimensions that interact with each other to form entangled networks and increase the viscosity of the aqueous media. Gums also adsorb at the interface of oil and water in emulsions and generate rupture-resistant multilayers that surround dispersed globules and act as mechanical barriers to impede coalescence [4]. Acacia, tragacanth, and guar gums have been effectively used as emulsifying agents [5]. They, however, remain costly to import, making it imperative to identify and assess cheaper and readily available local gums with similar emulsifying properties. Local gums would reduce the costs of importation and consequently, the cost of pharmaceutical emulsions. It would also represent a readily available and renewable source of emulsifying agents and reduce the time lag required to take receipt of imported emulsifiers [6].

*Melia azedarach* (MA) is a wooden plant that produces copious amounts of tasteless, clear to dark amber-colored gum [7]. The gum is a capable binder and disintegrating agent in tablets. This study also revealed that the gum has good swelling and flow properties. The good swelling property implies that the gum may readily interact with water to form the gel network to stabilize emulsions. However, the potential of this gum as an emulsifying agent has not been investigated. Local availability and the merits of natural polymers make it prudent to assess the emulsifying properties of MA gum. This study seeks to purify, characterize, and investigate the suitability of MA gum as an emulsion stabilizer.

## 2. Materials and Methods

### 2.1. General Materials

Food-grade olive oil manufactured by Bell Sons and Co. was purchased from a local pharmacy. The Department of Pharmaceutics, Kwame Nkrumah University of Science and Technology (KNUST), provided acacia gum, all growth media, and other reagents.

### 2.2. Collection, Extraction, and Purification of the *Melia azedarach* Gum

The crude gum was sourced as exudates from the incised trunks of the *Melia azedarach* tree found in the forests of Kwahu Asakraka in the Eastern Region of Ghana. The gum was harvested between March and May of 2022. The tree was identified and authenticated by the Herbal Medicine Department, KNUST curator. Extraneous materials were removed and the gum was dried at 50°C until sufficiently brittle. The light-grade gum was separated from the darker grade and the light-grade gum was pulverized using the miller (SK-1, RETSCH, Germany). The crude gum (100 g) was mixed with 200 ml of distilled water and left for 24 hours with intermittent stirring. The formed mucilage was filtered twice using a calico strainer. Ethanol (96%, 350 mL) was then added to precipitate the gum. The precipitated gum was washed with diethyl ether twice and dried in a hot air oven (Gallenkamp Oven, 300 Plus Series, Germany) at 40°C for 24 hours. The dried gum was powdered and sifted through sieve number 80 and stored in airtight packages [8].

### 2.3. Assessment of the Percentage Yield

The weight of the dry gum before purification, W_0,_ and the weight of the dry purified gum, W_1_, were determined using the analytical balance (Sartorius, LE623P, Germany). The percentage yield was estimated using the following equation:

(1) $$\begin{array}{c} \text{percentage yield}=\left(\frac{W1}{\text{Wo}}\right)\times 100\%. \end{array}$$

### 2.4. Evaluation of the Physicochemical Properties of the *Melia azedarach* Gum

#### 2.4.1. Macroscopic and Organoleptic Properties of the Crude *Melia azedarach* Gum

The color, odor, and taste were determined using organoleptic approaches. Morphology and general appearance were also visually assessed and recorded.

#### 2.4.2. Swelling Index of the Pure *Melia azedarach* Gum

The MA gum (1 g) was weighed into a 50 ml measuring cylinder and distilled water was added to make a 25 mL dispersion. The cylinder was covered, its content mixed, and allowed to stand undisturbed for 24 hours. The final volume of the dispersion was measured and recorded [9]. Equation (2) was used to calculate the swelling index as

(2) $$\begin{array}{c} \text{swelling index}=\left[\frac{\left(V_{f}-V_{o}\right)}{V_{o}}\right]\times 100\%, \end{array}$$

where *V*_*f*_ and *V*_*o*_ are the final and initial volumes of gum, respectively.

### 2.5. Proximate Analysis of the Purified *Melia azedarach* Gum

#### 2.5.1. Assessment of the Moisture Content of the Purified *Melia azedarach* Gum

The MA gum (1 g) was weighed into a previously dried and weighed porcelain crucible. The two were dried in a hot air oven at 105°C for 5 hours, allowed to cool, and weighed. The weight of the dried gum was obtained by deducting the weight of the crucible from the total weight of the gum and the crucible obtained after drying [9], and the moisture content was calculated as indicated in the following equation:

(3) $$\begin{array}{c} \text{moisture content}=\left[\left(\frac{W_{t}B-W_{t}A}{W_{t}B}\right)\right]\times 100\%, \end{array}$$

where *W*_*t*_*B* and *W*_*t*_*A* are the weight of the gum before drying and the weight of the dry gum, respectively.

#### 2.5.2. Mineral Content of the Purified *Melia azedarac*h Gum

A quantity of 5 mL of HNO_3_ (69%) was added to MA gum (1 g) in a 250 mL beaker. The sample was heated in a fume chamber at 100°C and mixed with perchloric acid (70%, 15 mL) and digestion was sustained until the solution was clear. The solution was cooled, made up to 100 mL with distilled water, and boiled for 10 minutes. The hot mixture was strained into a 100 mL volumetric flask through filter paper and made up to volume [10]. An atomic absorption spectrophotometer (novAA 400P, Analytik Jena GmbH, Germany) was used to determine the concentration of metal ions in the sample, using a regression equation of a linear calibration analysis. The sample was analyzed in duplicates.

#### 2.5.3. Determination of Protein, Fat, and Carbohydrate Content

MA and acacia gum compositions were determined using official methods [11]. The Kjeldahl method (Tecator^TM^ Kjeltec System) was used to assess the protein content. Fat content was determined by Soxhlet extraction using petroleum ether as a solvent. Carbohydrate content was calculated by subtracting the sum of the moisture content, total ash, protein, fat, and metal elements from 100%. Determinations were performed in triplicates.

#### 2.5.4. Ash Values

The gum (2 g) was placed in a crucible of known weight (W1) and heated in a muffle furnace at 550°C for 4 hours. The crucible and content were allowed to cool and maintained at a temperature below 200°C and then maintained at that temperature for 20 minutes. The crucible with the now ashen sample was cooled to 25°C and weighed (W3). For acid-insoluble ash, a mixture of 25 ml of HCl and hot distilled water (2 : 5) was used to quantitatively transfer the ash from the total ash experiment into a beaker, stirred with a glass rod, and heated for 5 minutes. The acid solution was filtered through an ashless filter paper. The filter was washed with hot water until the washings were acid-free to litmus paper. The filter paper was left to drain any residual fluid and placed in a crucible of known weight. The filter paper and crucible were dried and combusted in a furnace at 600°C. The crucible was cooled and weighed [11]. The ash content was calculated by using the following equation:

(4) $$\begin{array}{c} \text{percentage total ash}=\left[\left(\frac{W_{3}-W_{1}}{W_{2}}\right)\right]\times 100\%. \end{array}$$

The same equation was used to calculate the percentage of acid-insoluble ash. *W*_1_ is the weight of the empty crucible, *W*_2_ is the weight of the sample, and *W*_3_ is the weight of total ash or acid-insoluble ash and crucible depending on the parameter being estimated.

### 2.6. Assessment of the Flow Properties of the *Melia azedarach* Gum

#### 2.6.1. Bulk Density Measurements

A quantity of 20 g of the purified gum was weighed into a 100 mL measuring cylinder and the initial volume was recorded. The cylinder was tapped about 50 times to a constant final volume. The procedure was repeated twice. The weight of the gum and the initial and final volumes were used to calculate the fluff and tapped densities from which Hausner's ratio and Carr's index were deduced using the following equations, respectively [9]:

(5) $$\begin{array}{c} \text{Hausner ratio}=\frac{\text{tapped density}}{\text{fluff density}}, \end{array}$$

(6) Carr's index=tapped density – fluff densitytapped density×100%,

#### 2.6.2. Angle of Repose

The MA gum (10 g) was poured through a funnel clamped at a height that allowed a cone to be formed whose tip was just beneath the orifice of the funnel. The gum was allowed to flow freely onto a horizontal surface to form the cone. The diameter of the base of the cone was measured and the radius was taken as half of the diameter. The angle of repose was determined as *θ* by using the following equation:

(7) $$\begin{array}{c} \text{Tan}\theta =\frac{h}{r}, \end{array}$$

where *h* represents the height of the cone from the horizontal surface and *r* is the radius of the cone formed [9].

### 2.7. Determination of the Microbial Quality of the *Melia azedarach* Gum

MA gum mucilage (1%w/v) was prepared using sterile water. This mucilage (1 mL) was inoculated into previously sterilized growth media. The inoculated agar was allowed to stand for about 30 minutes, inverted, and incubated at 37°C for 48 hours (25°C in Sabouraud agar). Controls were also set up by incubating the growth media without the gum under the same conditions. The selective media used were observed for the presence or absence of characteristic microorganisms.

### 2.8. Phytochemical Screening of the *Melia azedarach* Gum

The presence or absence of phytochemical constituents was determined using the methods described by Shaikh and colleagues [12]. The target phytoconstituents tested include saponins, coumarins, phytosterols, tannins, flavonoids, triterpenoids, alkaloids, and glycosides.

### 2.9. FTIR Analysis of the *Melia azedarach* Gum

The FTIR uses the attenuated total reflectance (ATR) with a diamond crystal. The diamond crystal was cleaned with isopropanol and a background scan was quickly taken. The sample was placed directly on the crystal and the pressure gauge was applied to ensure maximum contact. The sample was then scanned between wavelengths of 4000 and 400 cm^−1^to generate the spectrum.

### 2.10. Preparation of Emulsions

Amounts of MA and acacia gum required to prepare emulsions containing 2.5%, 5%, 7.5%, 10%, 12.5%, and 15%w/v of gum were weighed and distilled water was added to make a 35 mL dispersion. This was mixed with a magnetic stirrer for about 2 hours at room temperature and stored at 4°C for 18 hours to ensure complete hydration [13]. A quantity of 15 mL of olive oil was measured and added dropwise to the dispersions while mixing with a magnetic stirrer. The mixture was then homogenized with the ULTRA-TURRAX homogenizer (T25, IKA, Germany) at 22000 rpm for 3 minutes to obtain the final emulsion. This procedure was used for the other gum dispersions [14].

### 2.11. Assessment of Emulsions

#### 2.11.1. Determination of Emulsion Type

The emulsion type was determined using the dilution test. Two 5 mL samples of each emulsion prepared with both gums were measured and transferred into a test tube. Excess water was added to each sample and observed for miscibility or otherwise [15].

#### 2.11.2. Analysis of Average Droplet Size, Zeta Potential, and Polydispersity Index

The samples were analyzed using Zetasizer (Nano ZS, Malvern Instruments, UK). Deionized water was used to perform 1 in 100 dilution of the emulsion. The diluted sample was placed in a cuvette and analyzed at 25°C. To determine the zeta potential, a cuvette fitted with electrodes was used. The tests were performed in triplicates. The refractive index values used for the dispersed and continuous phases were 1.47 and 1.33, respectively [13].

#### 2.11.3. Viscosity of Emulsions

The viscosity of emulsions was determined using the viscometer (Brookfield LVT, SN: 8401183, USA) at room temperature. The determination was performed at a rotation speed of 3, 6, and 12 rpm using spindle number 1. The viscosities were measured triplicate in cP.

#### 2.11.4. Emulsion Capacity and Emulsion Stability

The prepared emulsions were transferred into falcon tubes and the initial height of the emulsion was noted. Emulsions were centrifuged at 1200 *g* for 5 minutes immediately after preparation to determine the emulsion capacity. For emulsion stability, the emulsions were initially heated at 80°C for 30 minutes before centrifugation. The emulsions were centrifuged at 1200 *g* for 5 minutes using the centrifuge (Heraeus Biofuge Primo, Thermo Fischer Scientific, USA) and then the final height of the emulsion after centrifugation was noted [9, 16]. The equation is as follows:

(8) $$\begin{array}{c} \frac{\text{emulsion capacity}}{\text{emulsion stability}}=\left[\frac{\text{final height of emulsion after centrifugation}}{\text{total height of emulsion}}\right]\times 100\%. \end{array}$$

#### 2.11.5. Creaming Stability of Emulsions

Emulsions (10 mL each) were transferred into test tubes, capped and stored at room temperature, and observed for instabilities such as creaming and cracking. The emulsions' initial height was recorded to assess the creaming index. Creaming in the emulsions was assessed immediately after preparation and then on days 3, 7, and 14. The emulsion was separated into two layers one of which was creamy and another was straw-colored (serum). The stability of the emulsions on storage was determined by calculating the creaming index via the following formula [16]:

(9) $$\begin{array}{c} \text{creaming index}\left(\text{CI}\right)=\left[\frac{\text{final height of serum on storage}}{\text{total height of emulsion}}\right]\times 100\%. \end{array}$$

#### 2.11.6. Effect of Environmental Factors on Emulsions

The effects of environmental factors such as pH, electrolytes, and temperature on the average particle size, zeta potential, and creaming index were investigated for the emulsions with the most desirable properties. Acacia and MA gum emulsions containing 15%w/v of gum were selected and divided into sufficient portions for subsequent analysis. The emulsions selected were adjusted to contain 0.1 M, 0.2 M, and 0.3 M KCl and 0.1 M, 0.2 M, and 0.3 M CaCl_2_. To determine the effects of temperature, emulsions were exposed to temperatures of 25–90°C. The pH of the MA and acacia emulsions was also adjusted to 2, 5, 7, and 10 using either 0.1 M hydrochloric acid or 0.1 M sodium hydroxide solutions as required. The effect of these factors on the particle size, zeta potential, and creaming index was determined using the previously described methods [13].

#### 2.11.7. Statistical Analysis

The results of the tests that were carried out on the MA gum and acacia gum emulsions were compared by one-way analysis of variance (one-way ANOVA) followed by the Brown–Forsythe test using the GraphPad Prism (version 9). A *p* value <0.05 indicates significant statistical differences.

## 3. Results and Discussion

### 3.1. Percentage Yield

The percentage yield from gum extraction may depend on factors such as season, geographical location, solvent choice, and extraction procedure [7]. The extraction process employed for this gum resulted in a percentage yield of 68.3% ± 0.2500, a value comparable to that obtained by Owusu and colleagues [7] for the same gum. This value is, however, higher than that obtained for other gums including *Albizia* with a yield of 39.38% [17], *Tamarindus indica* with a yield of 29.83% [18], and *T. cordifolia* gum with a yield of 54.96 ± 2.15% (w/w) [19]. The relatively high yield suggests that the extraction process and the solvents used were ideal for the MA gum.

### 3.2. Physicochemical Properties of the Melia azedarach Gum

#### 3.2.1. Macroscopic Properties of the *Melia azedarach* Gum

**Figure 1:**
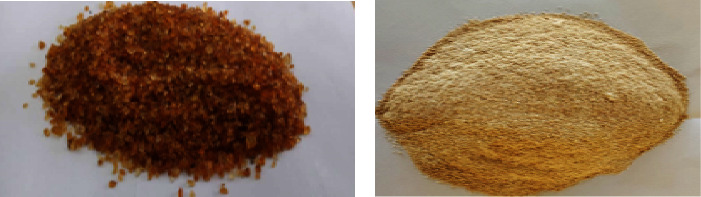
Crude (a) and purified (b) MA gum. The crude gum has a darker-brown color compared to the purified gum.

Physical and organoleptic properties such as color, taste, odor, shape, size, and hardness are influenced by environmental conditions, age of exudate, and treatment of gum after collection. The macroscopic characteristics of the crude gum are presented in Table 1. Commercially, the color of the gum is of a particular essence, and light colors are most desired. After purification, the crude MA gum had a yellowish-brown color and light grade (Figure 1).

The light-colored nature of the gum suggests that it might contain low levels of impurities. Gums are often expected to be tasteless and odorless or nearly so even though a few gums have been found to possess distinctive taste.

The purified MA gum was tasteless and odorless. This lack of a distinctive taste implies it may not interfere with such properties if used as an ingredient in pharmaceutical products.

The moisture content, total ash, water-soluble ash, and water-insoluble ash were based on the triplicate determination with the standard deviations as shown in Table 1.

#### 3.2.2. Swelling Index of the Purified *Melia azedarach* Gum

In emulsions, a gel network presence within the continuous phase limits instabilities induced by gravity or Brownian motion [20]. Higher swelling indices imply higher entrapment of water and improved formation of gel networks [18]. The swelling index of MA gum was 82.9% ± 0.46. The value although lower than that of *Albizia* (611.29 ± 4.07) gum [17] is considerably better than that of tamarind seed mucilage (7.6–21.196) [21]. The recorded value suggests that the gum is capable of interacting strongly with water molecules to form a gel network. Consequently, it implies that the gum may possess properties that aid in the stabilization of oil-in-water emulsions.

### 3.3. Proximate Analysis of the Purified *Melia azedarach* Gum

#### 3.3.1. Moisture Content in the *Melia azedarach* Gum

Moisture in a natural product usually affects chemical and flow properties and may enhance the growth of microorganisms. Economically, high moisture content is undesirable as determined weights may not be entirely attributable to the product itself and may be accounted for by moisture [17]. The moisture content of the gum was determined to be 9.28% ± 2.40. This is lower than the value (11.95%) reported [22] for acacia gum, the most established gum used as an emulsifier. The moisture content of the gum thus complies with this standard. This relatively low moisture content suggests a reduced susceptibility to chemical changes or decomposition by microbes and a reduced likelihood of clumping of dry gum powder. Economically, this is favorable as any determined weight of gum is highly attributable to the gum itself rather than any associated moisture.

#### 3.3.2. Mineral Content

The atomic absorption spectrophotometer was used to quantify elements in the gum. Lead, copper, cadmium, sodium, and mercury were either absent or were below the detection limits. Zinc (0.3736 ± 0.0018 mg/L), calcium (4.419 ± 0.2761 mg/l), iron (0.4475 ± 0.0255 mg/l), magnesium (11.24 ± 0.0335 mg/l), manganese (0.6387 ± 0.0226 mg/l), and potassium (0.2535 ± 0.0080 mg/L) were detected in quantities indicated in brackets. The elemental content values detected in this report are lower than those reported in other plant materials [23, 24]. The absence of heavy metals makes MA gum a promising candidate excipient in pharmaceutical and food products.

#### 3.3.3. Carbohydrate, Fat, and Protein Content

The protein content (1.02 ± 0.1556%) of the gum is higher than that of Persian gum (0.69%) and *ammoniacum* gum (0.3%) reported by Golkar and others [13] and Ebrahimi and colleagues [14], respectively. This value is, however, lower than what was observed for acacia gum (1.97 ± 0.3110%). The presence of proteins in polysaccharides may improve their ability to adsorb at oil-water interfaces, and that contributed to the enhancement of their emulsion-stabilizing properties [4]. The presence of protein in the MA gum thus projects its likelihood of being a stabilizer. The gum had a carbohydrate content of 87.561% compared to 87.453% for acacia. Golkar and colleagues [13] again reported that a high carbohydrate content confirms the purity of gums. As such, the high carbohydrate content recorded for the acacia and MA gum indicates that they are of good quality.

#### 3.3.4. Ash Values

Ash value is useful for identifying low-grade products. Total ash detects if plant products are mixed with materials such as soil and inorganic salts such as calcium oxalate. The acceptable range of total ash varies within wide limits and cannot be relied upon solely. The total ash for the gum is 1.2% ± 0.05. This value is relatively low in comparison to that detected for other gums such as *ammoniacum* gum (3.6%) by Ebrahimi et al. [14] and Persian gum (2.59%) by Golkar et al. [13]. The results indicate that the gum contains low amounts of inorganic materials, sand, and other soil components. The results, however, cannot be relied on completely in light of the previously stated demerit. The acid-insoluble ash evaluates the amount of ash that is insoluble in hydrochloric acid. It provides useful information regarding the quantity of inorganic material detected using total ash represented by material other than calcium oxalate. The acid-insoluble ash was determined to be 0.28% ± 0.0173. This represents a relatively small fraction of the total ash.

### 3.4. Flow Properties

Many industrial processes involve the movement of powder from one location to another using different methods. In all of these methods, the flow rate of the powder depends on the process design and significantly, the powder flow properties [25]. The angle of repose, Hausner's ratio, and Carr's index were determined to ascertain the flow properties of MA gum. A powder with excellent properties must possess an angle of repose, Hausner ratio, and Carr's index between 25 and 30°, 1.00 and 1.11, and 1 and 10%, respectively [25]. The results from this study returned values of 1.08 ± 0.02, 7.29 ± 1.83, and 30.35 ± 0.56 for Hausner's ratio, Carr's index, and angle of repose, respectively. These figures are consistent with excipients in some pharmaceutical industry activities including tableting and capsule-filling that require uniform flow of ingredients during the processes.

### 3.5. Microbial Quality

Fungi, bacteria, and viruses are ubiquitous in our environment. Some of these organisms produce malignant effects, whereas others remain relatively benign. Due to the former, materials included in foods and medicines should comply with set standards [26]. Food and pharmaceutical products should not contain *Salmonella* spp. and *E. coli* and the total aerobic count should be at most 10^5^ cfu/g and combined yeast and mold count be not more than 10^3^ cfu/g. In addition, bile-tolerant Gram-negative bacteria count should not exceed 10^3^ cfu/g [27]. The results returned a negative test for *Staphylococcus aureus*, *Salmonella* spp., *Pseudomonas* spp. and *Escherichia col*i. Viable aerobic fungi counts were 250 cfu/g and 40 cfu/g, respectively. This result shows that the gum complies with the standards set by the USP. This could be attributed to hygienic harvesting practices, efficient purification procedures, and storage conditions. MA gum may be incorporated into products with confidence that it will not compromise safety by introducing excessive amounts of potentially pathogenic organisms.

### 3.6. Phytochemical Screening

The phytochemical constituents present include flavonoids, tannins, glycosides, and saponins. Except for flavonoids, these are the same as those found in earlier research [7]. The differences observed may be attributed to the differences in the age of the plants, the season of harvests, and environmental conditions [28]. Tannins and glycosides possess antioxidant properties; in addition, glycosides have some antimicrobial activity [29]. The gum may have the capacity to confer preservation on products that may be incorporated. The amphiphilic saponins present confer surface activity on the gum [30] and make it a potential emulsion stabilizer.

### 3.7. FTIR Analysis of the *Melia azedarach* Gum

**Figure 2:**
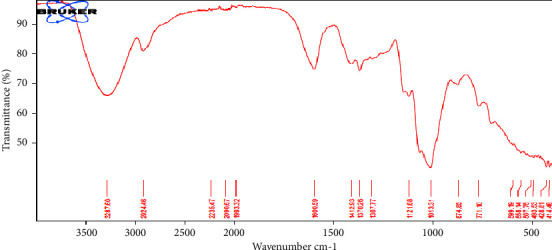
FTIR spectrum of the MA gum.

The Fourier-transform infrared (FTIR) spectroscopy is a good tool for identifying functional groups present in materials [31]. It has helped in the identification of materials and the detection of adulterations. FTIR analysis was performed to provide a fingerprint for MA gum as shown in Figure 2. The spectrum showed a medium stretch at 3287.60 cm^−1^, which indicated the presence of an O––H. In addition, at 2924.46 cm^−1^, an alkane C––H stretch was observed, highlighted by the occurrence of a medium C––H bend at 1370.27 cm^−1^ and a medium C––C stretch at 1412.93 cm^−1^. Equally, an aromatic C––C stretch was observed in the 1600 cm^−1^ region. This data can be used to aid the easy identification of the gum.

### 3.8. Evaluation of Formulated Emulsions

#### 3.8.1. Appearance, pH, and Type of Emulsions

**Figure 3:**
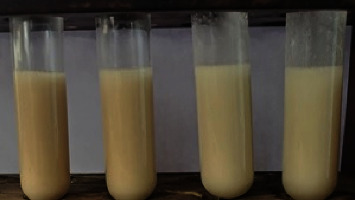
Sample of emulsions formulated with the *Melia azedarach* gum.

All emulsions (Figure 3) were creamy and appeared stable except for the ones containing 2.5%w/v gum, which began to cream minutes after formulation. The pH was assessed to predict the stability of the emulsions and how they may affect mucosae. The pH of the emulsions was in the acidic range (5.2−4.2). It is reported that the prescribed pH for formulations designed for oral administration ranges from 5 to 8 [6]. The acidic nature of the emulsions suggests that they may irritate the oral mucosa. Thus, the pH will have to be adjusted to near-neutral to reduce the tendency of mucosal irritation. All emulsions were of the oil-in-water (o/w) type. This is in line with the fact that hydrophilic gums favor the formation of oil-in-water emulsions.

All the emulsions were creamy and homogenous.

#### 3.8.2. Viscosity of Emulsions

During storage, viscosity is an important factor in the stability of emulsions. High viscosity reduces the coalescence of oil droplets and improves stability [13]. Increasing gum concentration increased the viscosity of the emulsion, which is consistent with previous reports [32]. The viscosities of the MA gum emulsions were higher than those of acacia gum at all concentrations. This implies that the MA gum forms a relatively higher proportion of gel networks making it more efficient at slowing down droplet aggregation. The viscosity of the emulsions decreased with increasing shear speed, which depicts shear-thinning behavior. Applying sufficient force then will reduce the viscosity of these emulsions, making them easier to pour from containers and to apply to the skin if used in topical creams.

#### 3.8.3. Emulsion Capacity (EMC), Emulsion Stability (EMS), and Creaming Index

The emulsifying capacity assesses if an agent is capable of forming emulsions. The emulsion stability characterizes the destabilization process as a function of time [18]. The emulsion capacity and stability were enhanced, whereas creaming was reduced when the concentration of the gums was increased. The stability of emulsions containing the MA gum was higher than those containing the acacia gum. This is attributable to the higher viscosity that reduces the tendency for the droplets to collide. Higher emulsion capacity and stability do not prove that MA gum possesses a higher surface activity than acacia since the particle size of emulsions produced with MA gum increased as concentration was increased. Huang et al. [33] made similar observations for emulsions prepared with various gums that did not contain an emulsifying agent. The observation in this work may be attributed to a relatively lower protein quality in the MA gum. Proteins improve the surface activity of gums such as acacia, guar, and fenugreek [34]. Jafar and colleagues also reported the surface tension reduction by gum from *Ferula gummosa* [35]. Thus, it may be reasonable to assume that MA gum may not possess similar surface activity as acacia gum and other gums [35] but contributes to the stability of the emulsion by forming a gel network that traps oil droplets. Creaming reduced as the concentration of gums increased. As the days went by, creaming increased progressively in the emulsions with lower concentrations (5 and 7.5%). For the emulsions with concentrations of 10%, 12.5%, and 15%, no creaming was experienced after 7 days of storage. These observations are consistent with those of Koocheki and coworkers [36], where increased gum concentration was found to retard creaming. The acacia emulsions creamed earlier than their MA gum counterparts of similar concentration.

#### 3.8.4. Effect of the Concentration of Gum on the Zeta Potential and Average Droplet Size of Emulsions

**Figure 4:**
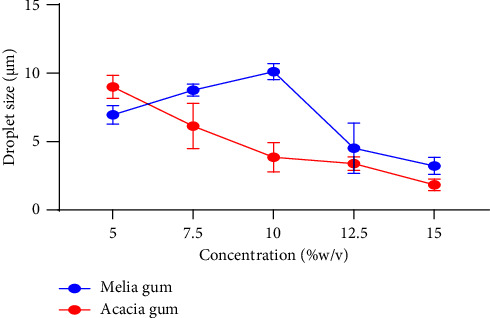
Effect of gum concentration on droplet size.

Smaller droplets improve the stability of emulsions. In the acacia gum emulsions, there was a significant (*p* value <0.0001) decrease in droplet size as the concentration of the gum increased from 5 to 15%, with the smallest droplet size (1.837 ± 0.420 *μ*m) being observed at 15%w/v (Figure 4).

The determinations were performed in triplicates. The MA gum emulsion seems to have a relatively larger average droplet size compared to that of the acacia gum.

Previous studies have reported that 14% of acacia gum is sufficient for covering the total area of the interfacial region of an oil-in-water emulsion [37]. For the MA gum, the particle size increased when the concentration of gum was increased from 5% to 10% w/v. However, emulsions containing more than 10% MA gum had smaller particle sizes (4.520–2.791 *μ*m) with the 15% MA emulsion having the least droplet size (2.791 ± 0.694 *μ*m). Gums stabilize emulsions by increasing viscosity and inducing steric stabilization [34, 36], which could account for the observed results.

A modest increase (65–161.25 cP) in viscosity was observed for emulsions containing an increasing amount (5–10%) of MA gum. However, high increments (161.25–483.75 cP) are seen as the concentration rises from 10–15%. Larger particles at lower concentrations could be observed because the relatively low viscosities are insufficient to hinder aggregation. However, as the viscosity increases significantly, the movement of particles is sufficiently slowed, preventing them from aggregating into larger particles.

**Figure 5:**
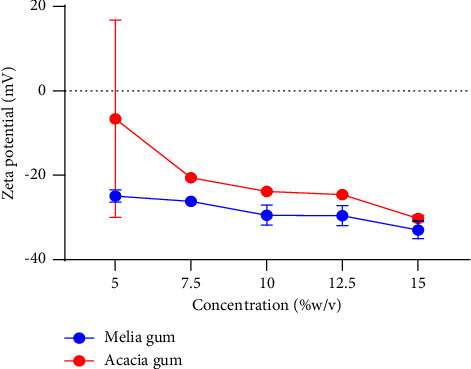
Effect of gum concentration on the zeta potential of emulsions.

The efficiency of MA gum at reducing droplet size compared to acacia gum was determined by assessing Z-average. Steric stabilization tends to occur when a high enough amount of gum is available to cover the surface of oil droplets [34]. The gum may induce steric repulsion between droplets close to one another and prevent them from aggregating. This phenomenon probably occurs at concentrations greater than 10% for MA gum. It was determined that both gums resulted in negative zeta potentials which increased as the concentration of the gums increased (Figure 5). The highest (acacia: −30.45 mV; MA gum: −32.867 mV) zeta potential for both gums was observed at 15%w/v.

The zeta average of the emulsion droplets appeared to reduce when the gum concentration was increased.

The absolute value of zeta potential at 15% for both gums is higher than 30 mV (−30.45 mV for acacia and −32.867 mV for MA gum) and the limit for significant droplet stabilization by electrostatic repulsion [38]. The low particle size and sufficient zeta potential produced at 15% gum concentration suggest that the gum may have emulsion-stabilizing properties. Acacia, however, produced smaller droplets as compared to MA gum at 15% concentration where both gums had the highest zeta potential. The presence of some protein and the reduction of particle size by MA gum confirmed that it may be a promising candidate for the stabilization of emulsions [39].

#### 3.8.5. Effect of Electrolytes on the Formulated Emulsions

**Figure 6:**
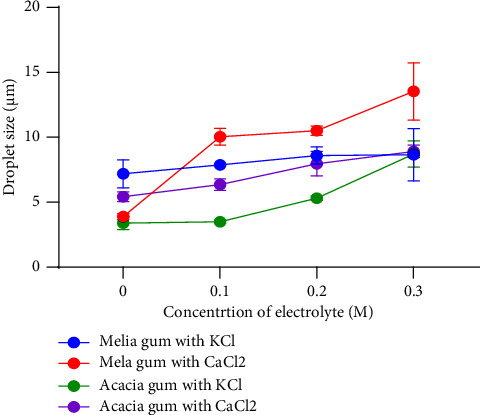
Effect of electrolytes on the average droplet size.

Electrolytes may affect zeta potential and produce a system where attractive forces exceed repulsive forces depending on the nature of the electrolytes. This phenomenon promotes the aggregation of dispersed phase particles to form larger droplets [40]. The effects of KCl and CaCl_2_ on the particle size and zeta potential of emulsions were examined. It was observed (Figure 6) that for both gums, an increase in KCl concentration caused a significant (acacia, *p* value <0.0001; MA, *p* value −0.4342) increase in particle size.

### 3.9. Electrolytes Cause an Increase in Droplet Size due to a Drop in Zeta Potential

Increasing electrolyte concentration shrinks the electrical double layer that surrounds the dispersed globules, thus decreasing the zeta potential. Attractive forces exceed repulsive forces and particles aggregate through flocculation [34]. This accounts for the increase in droplet size seen. These results are in line with what has been found by other researchers [13, 14]. Calcium chloride (CaCl_2_) produced a more pronounced effect compared to that of KCl, which could be a result of its comparatively higher ionic strength. Creaming increased when the concentration of electrolytes was increased. Emulsions containing acacia creamed at a faster rate than those with MA gum. The rate and extent of creaming are directly proportional to particle size but indirectly proportional to the viscosity of the emulsion. The increase in creaming index can thus be attributed to the increased particle size resulting from increasing electrolyte concentration. However, the slow rate of creaming associated with MA gum emulsions may be because they are more viscous than acacia emulsions.

#### 3.9.1. Effect of Temperature on Emulsions

Emulsions are exposed to various temperatures during production, sterilization, and storage. The heating of emulsions enhances hydrophobic interactions and may usually lead to aggregation of droplets. The stability of MA and acacia gum emulsions to droplet aggregation after heating (25 to 90°C) was assessed. It was determined that the temperature had no significant effect on the droplet size of acacia (*p* value = 0.0774) and MA gum (*p* value >0.9999) emulsions, an observation similar to that reported by Golkar et al. [13]. These results suggest that the gum will be stable when the emulsion is heated. Emulsions that are stabilized with hydrocolloids are not affected by denaturation at high temperatures [41], and this may have accounted for the reason why droplet aggregation caused by heating had little impact on oil droplet size. Similar results [42] were reported where orange oil-in-water emulsions remained stable to droplet aggregation after heat treatment.

#### 3.9.2. Effect of pH on Emulsions

The pH of gum solutions was adjusted to 2, 5, 7, and 10 to determine the effect of varying pH on emulsion properties. It was observed that the droplet size of emulsions stabilized by both gums increased (acacia: 2.792–6.080 *μ*m, MA gum: 3.159–9.068 *μ*m) significantly (acacia, *p* value 0.0006; *Melia* gum, *p* value –0.0356) as pH varied from 10 to 2. The zeta potential was again found to be negative for emulsions stabilized by both gums and increased from −20.3 mV to −31.5 mV and −22.3 mV to–31.83 mV for acacia and *Melia* gum emulsions, respectively, within the pH range of 2–10. As pH decreases from 10 to 2, the negative charges are shielded by the positive charges of the medium to produce acidic pH [42]. This leads to a decrease in electrostatic repulsion, which results in the reduction of zeta potential. Droplet aggregation increases and leads to the formation of larger globules. The increase in particle size may negatively impact the stability of the emulsions because smaller sizes may be relatively more stable than those with larger droplets. The stability of emulsions may be affected by pH. From the results obtained, manufacturers must consider the effect of different ingredients on the pH of the MA gum emulsions.

## 4. Conclusion

This study assessed the physicochemical properties of the *Melia azedarach* gum and compared its emulsifying properties with those of acacia. A yield of 68.3% was obtained and the gum had low ash values, good flow properties, and desirable microbial content. Emulsions containing the acacia gum had lower droplets than those of the MA gum. MA gum, however, produced emulsions that were less susceptible to creaming and had better emulsion capacity. Both gums produced similar effects when emulsions were exposed to environmental stresses. The findings in this study show that the MA gum may be used to stabilize emulsions with low viscosity either alone or in combination with other emulsifiers. The authors are currently investigating this gum for other potential pharmaceutical applications.

## Figures and Tables

**Table 1:** Macroscopic and physicochemical properties of the MA gum (*R* = 3, where applicable).

Parameter	*Melia azedarach gum*
Taste	Tasteless
Color	Yellowish brown
Surface appearance	Glassy and smooth
Shape	Irregularly round
Moisture content (%w/w)	9.28 ± 2.4000
Total ash (%w/w)	1.20 ± 0.0500
Acid-insoluble ash (%w/w)	0.28 ± 0.0173
Water-soluble ash (%w/w)	0.70 ± 0.0500

## Data Availability

The information that justifies the investigation's findings can be found in the publication and is also obtainable upon request from the corresponding author.
